# Combining the Sterile Insect Technique with *Wolbachia*-Based Approaches: II- A Safer Approach to *Aedes albopictus* Population Suppression Programmes, Designed to Minimize the Consequences of Inadvertent Female Release

**DOI:** 10.1371/journal.pone.0135194

**Published:** 2015-08-07

**Authors:** Dongjing Zhang, Rosemary Susan Lees, Zhiyong Xi, Jeremie R. L. Gilles, Kostas Bourtzis

**Affiliations:** 1 Insect Pest Control Laboratory, Joint FAO/IAEA Division of Nuclear Techniques in Food and Agriculture, Vienna, Austria; 2 Sun Yat-sen University—Michigan State University Joint Center of Vector Control for Tropical Diseases, Zhongshan School of Medicine, Guangzhou, Guangdong Province, China; 3 Department of Microbiology and Molecular Genetics, Michigan State University, East Lansing, Michigan, United States of America; University of Camerino, ITALY

## Abstract

Due to the absence of a perfect method for mosquito sex separation, the combination of the sterile insect technique and the incompatible insect technique is now being considered as a potentially effective method to control *Aedes albopictus*. In this present study first we examine the minimum pupal irradiation dose required to induce complete sterility in *Wolbachia* triple-infected (HC), double-infected (GUA) and uninfected (GT) female *Ae. albopictus*. The HC line is a candidate for *Ae. albopictus* population suppression programmes, but due to the risk of population replacement which characterizes this triple infected line, the individuals to be released need to be additionally irradiated. After determining the minimum irradiation dose required for complete female sterility, we test whether sterilization is sufficient to prevent invasion of the triple infection from the HC females into double-infected (GUA) populations. Our results indicate that irradiated *Ae. albopictus* HC, GUA and GT strain females have decreased fecundity and egg hatch rate when irradiated, inversely proportional to the dose, and the complete sterilization of females can be acquired by pupal irradiation with doses above 28 Gy. PCR-based analysis of F_1_ and F_2_ progeny indicate that the irradiated HC females, cannot spread the new *Wolbachia w*Pip strain into a small cage GUA population, released at a 1:5 ratio. Considering the above results, we conclude that irradiation can be used to reduce the risk of population replacement caused by an unintentional release of *Wolbachia* triple-infected *Ae. albopictus* HC strain females during male release for population suppression.

## Introduction

*Aedes albopictus* is a competent vector of several severe arthropod-borne diseases including dengue fever, yellow fever and chikungunya [1–3]. Conventional control methods, such as those relying on insecticides, copepods, larval source reduction and community participation, are not sufficient to keep this species’ population density below the epidemic risk threshold [4,5]. Consequently, there is a crucial need for innovative and complementary means to control this mosquito species, such as the sterile insect technique (SIT) and the incompatible insect technique (IIT), as standalone approaches or used in combination [6–9].

SIT or IIT is based on mass-rearing of the target species, sterilization (by irradiation or infection with *Wolbachia pipentis*) and continuous release of the sterile insects into the target population [10]. The released sterile males mate with wild females, which will lead to a decrease in reproductive potential of the mated females, and with sufficient releases the target population should be suppressed [10]. Some successful examples of SIT programmes are the eradication of the New World screwworm *Cochliomyia hominivorax* Coquel from the USA [11], the eradication of the tsetse fly, *Glossina austeni* from the island of Unguja [12] and the eradication of the melon fly, *Bactrocera cucurbitae* from the Okinawa islands, Japan [13]. It has been shown that that the efficiency and cost-effectiveness of SIT programmes significantly increase if they are based on male-only releases [14]. IIT is also based on the same principles like SIT with the key factor being male-only releases. It is worth noting that the first successful application of IIT achieved eradication of the target population of filariasis vector *Culex pipiens* in Burma [15]. IIT is based on the mechanism of *Wolbachia*-induced cytoplasmic incompatibility (CI) which can be either unidirectional or bidirectional [16]. In most species, unidirectional CI is expressed as embryonic lethality in crosses between infected males with females which are either uninfected or infected with an additional *Wolbachia* strain. Bidirectional CI is expressed in crosses between males and females infected with different *Wolbachia* strains.

For mosquito SIT or IIT, there are many aspects requiring improvements to move to the operational level; however, the most critical step is sex separation [17]. Unlike agricultural insect pests, any released female mosquitoes are capable of biting people and of disease transmission [17]. Availability of an efficient and robust genetic sexing strain (GSS), like the GSS developed for SIT programmes for the Mediterranean fruit fly *Ceratitis capitata* (Vienna 8 GSS), would revolutionize mosquito SIT applications [17,18]. A GSS is not yet available for *Ae*. *albopictus*, but the difference in pupal body size between males and females means that the majority of the female pupae can be separated from the males through a Fay-Morlan separator [19,20]. The percentage of female contamination depends on the expertise of the operator and how the separation is done; selection of smaller individuals reduces the female contamination but at the same time results in loss of larger number of males. In large scale operations, millions of male adult mosquitoes are released each time and even a small percentage of residual females would mean that thousands of females are released, which may reduce the effectiveness of population suppression, and the release of potential disease vectors would not be acceptable. In addition, for mosquito IIT the accidental release of females infected with an additional *Wolbachia* strain with respect to the wild population may result in the replacement of the targeted population instead of the intended suppression [7–9]. The successful IIT programme in Burma depended on visual sorting of released individuals to safeguard against any accidental female release [15]. Manual inspection is unlikely, however, to be efficient or sustainable during the mass-rearing of hundreds of millions of mosquitoes required for large scale application [8].

In order to eliminate the risk of population replacement while aiming for suppression, a strategy combining SIT with IIT was proposed for the first time in *Culex pipiens molestus* [6] and was recently tested in a pilot trial in *Ae*. *polynesiensis* [8]. By combining SIT with IIT, the sterility of released males would be ensured by both irradiation and *Wolbachia* infection, while the irradiation would have the added benefit of female sterility [7–9].

Past studies have shown that in many insect species females produce few or no progeny after exposure to an adequate radiation dose [6,8,21–28]. Abdel and colleagues found that the number of developing oocytes of ovaries and the length of the ovariole decreased in *Plodia interpunctella* after exposure to more than 50 Gy of gamma irradiation [26], which could also explain the loss of fertility in other species. For females of different mosquito species, full sterility (defined by lack of egg lay or a null egg hatch rate) is acquired at different irradiation doses. For example, complete sterilization of *Cx*. *pipiens molestus* females requires a higher dose (70 Gy) than for *Cx*. *quinquefasciatus* females (60 Gy) using a ^60^Co radiation source [6]. No egg production is observed in *Ae*. *aegypti* females when they are exposed to 50 Gy of gamma radiation [29] while *Ae*. *polynesiensis* females are completely sterile after exposure to 40 Gy electron beam radiation [8]. In *Ae*. *albopictus*, egg production drops off sharply in females exposed to >20 Gy gamma radiation [30] and no progeny are produced when the dose of either gamma ray or X ray radiation is >40 Gy [30,31]. However, the minimum optimal dose for complete sterilization of female *Ae*. *albopictus*, which would at the same time have no effect on male mating competitiveness, remains unknown.

It was recently shown that there was no significant difference in fitness between the triple *Wolbachia*-infected *Ae*. *albopictus* HC strain (*w*AlbA, *w*AlbB, and *w*Pip), the double *Wolbachia*-infected GUA strain (*w*AlbA and *w*AlbB) and the aposymbiotic GT strain. All strains have a highly similar (>98%) genetic background originating from the Guangzhou region of China. Based on these results, Zhang and colleagues (2015) suggested that the *Wolbachia* triple-infected HC strain of *Ae*. *albopictus* is suitable for mass rearing and potential control programmes based on the combination of SIT and IIT [32]. Before such a strategy is applied to control natural populations of *Ae*. *albopictus*, the minimum irradiation dose to make females completely sterile should first be determined. In the present study, we determined the lowest X ray irradiation dose, applied at the pupal stage, required to produce complete sterility in female *Ae*. *albopictus* from three strains (HC, GUA and GT) with different *Wolbachia* infection status. Next, we assessed the ability of HC females to introduce and cause invasion by *Wolbachia w*Pip of cage populations of the GUA strain when they have first been irradiated at the pupal stage. Finally, our results are discussed in the context of combining SIT with IIT in a suppression programme to reduce the risk of accidental releases of triple *Wolbachia*-infected HC female *Ae*. *albopictus* into the wild, which might bring about population replacement rather than the intended population suppression.

## Materials and Methods

### Ethics statement

Research on mosquitoes (*Ae*. *albopictus*) does not require a specific permit according to directive 2010/63/EU of the European Parliament and the Council for the protection of animals used for scientific purposes. All mosquito strains used in the present study were maintained in the biosecure insectaries of the Joint FAO/IAEA Insect Pest Control Laboratory (IPCL), Seibersdorf, Austria. All the experiments were performed based on standard operating procedures in the IPCL. The blood used for routine blood-feeding of mosquitoes is collected in Vienna, Austria during routine slaughtering of pigs or cows in a nationally authorized abattoir (Rupert Seethaler, Himberg bei Wien) according to the highest possible standards and strictly following EU laws and regulations.

### Mosquito strains and rearing

The current study was conducted using the triple (*w*AlbA, *w*AlbB and *w*Pip) *Wolbachia*-infected HC strain, the double-(*w*AlbA and *w*AlbB) infected GUA strain and the *Wolbachia*-uninfected GT strain [32]. The F_12_ generation of GUA, the F_11_ generation of HC and the F_9_ generation of GT, since the establishment of the strains in the IPCL, were used for this experiment. All three strains were maintained and experiments conducted in a climate-controlled room at 27 ± 1°C, 80 ± 10% RH, and a photoperiod of 12:12 (L: D) h.

Adults were kept in standard 30 × 30 × 30 cm cages (BugDorm 1; MegaView, Taichung, Taiwan) and supplied *ad libitum* with a 10% sugar solution. Bovine or pig blood was provided to female mosquitoes (from 4 to 5 days post-emergence) three times per week. A plastic beaker with moist paper (white crepe paper, IF C140, Industrial Filtro S.r.l., Cologno Monzese, Italy) was put into each cage 48 hours after blood-feeding to collect eggs. Eggs were collected for 3 days and allowed to mature for 7 days, then were hatched by submerging in hatch solution (700 ml deionized water containing 0.25 g Nutrient Broth and 0.05 g Brewer’s Yeast [32] for 24 hours. Approximately 3,000 first-instar larvae (L_1_) were reared per plastic tray (40 × 29 × 8 cm) containing 1 liter of deionized water. The larvae were fed daily on IAEA2 diet as previously described [32,33]. Pupae were collected and placed in small plastic cups inside an adult cage for emergence.

### Irradiation of pupae

The RadSource RS 2400 X ray irradiator (Rad Source Technologies Inc., Suwanee, GA) was used for irradiation of *Ae*. *albopictus* pupae for all experiments. For irradiation groups of female HC, GUA and GT strain pupae (from 24 h to 36 h post pupation) were clustered in the centre of a plastic plate (50–70 pupae per plate) (see S1B Fig) by sieving them through a gauze strainer, and then rinsing them from the strainer on to the plates with deionized water. Any residual water in the plate could be removed by a pipette. Preliminary experiments where pupae were instead distributed across the whole plate (see S1A Fig) resulted in a low level of residual egg production (see S1 Table), highlighting the need for dosimetry experiments to determine the dose range across the field of radiation before irradiation of experimental material begins.

A piece of Gafchromic HC-810 film (1×1 cm, Ashland Specialty Ingredients, Wayne NJ) was placed centrally on top of the pupae. After irradiation, the Gafchromic film could be read by the Radiachromic reader (FWT-92D, Far West Technology, Inc., Goleta, CA) to determine the precise (± 2.0%) irradiation dose that pupae had received.

### Determining the minimum female sterilizing dose

Female HC, GUA and GT pupae were all irradiated at 0, 28, 32, 34, 38 or 40 Gy. After irradiation, 50 pupae of each strain were separately caged with 50 non-irradiated male pupae of the same strain. Four to five days post-emergence a blood-meal was provided daily for three days. Engorged females were placed into individual plastic tubes (2 cm diameter × 10 cm height, one female per tube) with 30 ml deionized water and a moist filter paper (6 × 6 cm) and left to oviposit for three days. Eggs were kept for seven days for maturation before adding the hatching solution and were allowed to hatch for 24 hours as described above. Fecundity was determined by counting the total number of eggs produced per female, while fertility was assessed by counting the number of eggs hatched out of the total number of eggs produced per female (the counting was performed under the stereomicroscope).

HC, GUA and GT strain females were all completely sterile after exposure to irradiation at 28 Gy. So, a further experiment was performed to determine the minimum irradiation dose required to induce complete sterility in HC strain females. HC strain female pupae were irradiated at 0, 23, 24, 26 and 28 Gy and female fecundity and fertility determined as described above.

### Effect of irradiation on female adult emergence rate

HC strain female pupae were irradiated at 0, 26, 28 and 40 Gy while GUA and GT strain female pupae were irradiated at 0 and 40 Gy. After irradiation, the pupae were placed into a plastic cage and left to emerge. Forty-eight hours later, dead pupae were removed and numbers recorded.

### Effect of irradiation on ovaries

To examine the effects of irradiation on the ovaries of HC, GUA and GT strains of *Ae*. *albopictus*, 15 pairs of ovaries from female adults (7–8 days old) of each strain irradiated as pupae were removed. In this experiment, HC strain female pupae were irradiated at 0, 26, 28 and 40 Gy while GUA and GT strain female pupae were irradiated at 0 and 40 Gy. The ovary length was measured as the length of the middle axis of the oval ovary. A digital image of each ovary (30 ×) was produced using a CC-12 camera mounted on a stereomicroscope, and measurements were performed using analysis B software (Olympus Soft Imaging Solutions GmbH, Munster, Germany). Non-irradiated females of each strain were also measured for control.

### Ability of irradiated HC females to spread *Wolbachia w*Pip into small cage GUA populations

**Fig 1:**
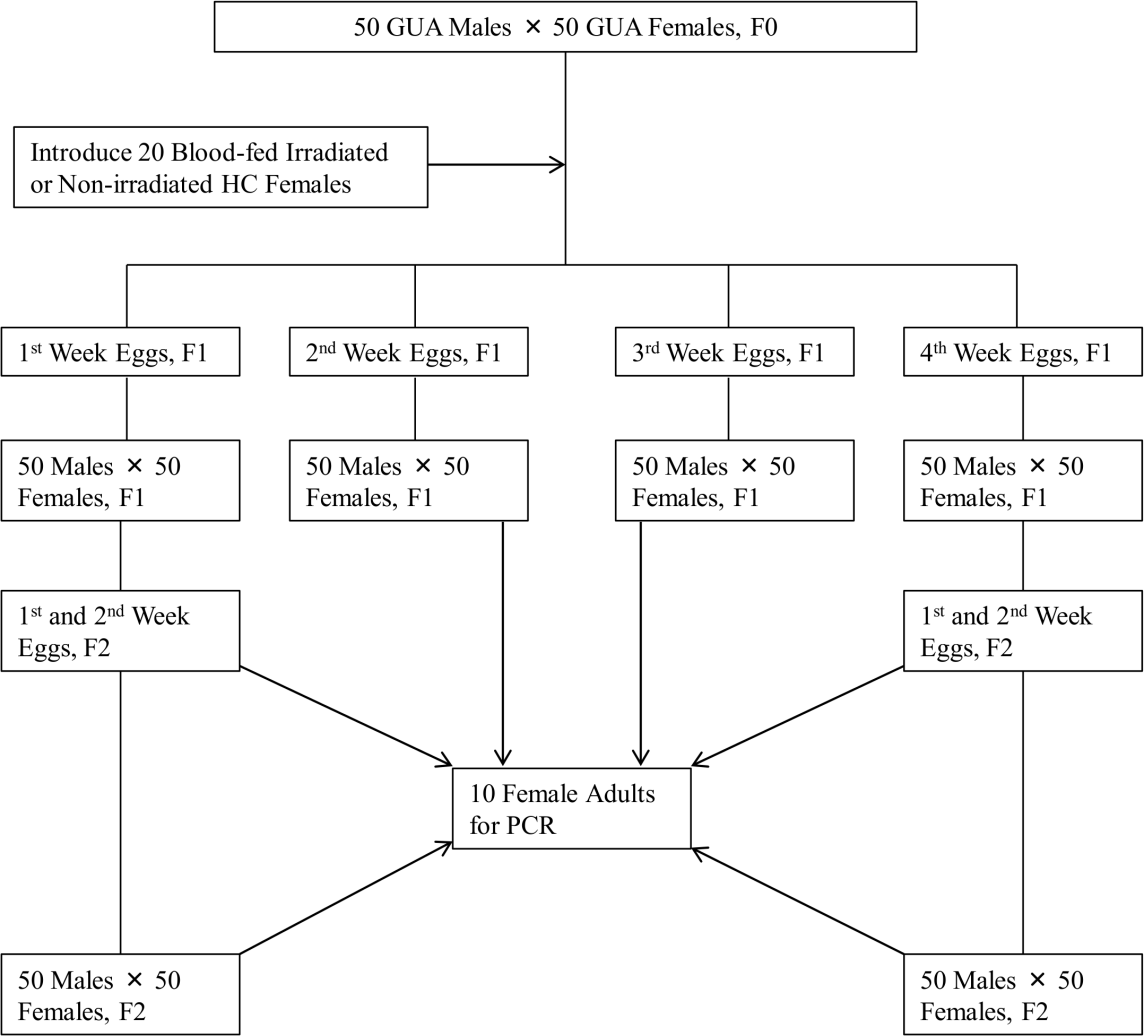
Experimental design to test the ability of irradiated and non-irradiated HC *Aedes albopictus* females to spread *Wolbachia w*Pip into small cage GUA populations.

We assessed the ability of irradiated triple-infected (*w*AlbA, *w*AlbB, *w*Pip) HC females to introduce and cause invasion by *w*Pip in a small cage (30×30×30 cm) population of the double-infected (*w*AlbA, *w*AlbB) GUA (wild type) strain. The experimental design is shown in Fig 1. Six GUA population were established, each consisting of 50 GUA females and 50 GUA males (2 day old F_0_ individuals). From three days after the establishment of the cage population, a blood meal was provided daily for the next three consecutive days. After the last blood meal of the 1^st^ week, i.e. day 7 post-emergence, 20 blood-fed irradiated HC females (28Gy) were added to each of the three (treatment) cages, representing an introduction of 1 HC female for every 5 GUA adults. Into each of the remaining three (control) cages 20 blood-fed but non-irradiated HC females were added. Oviposition sites were provided to the cage populations after the last blood meal. Blood meals were provided to all cages three times per week for the next three consecutive weeks. Eggs were collected for four weeks, allowed to mature for seven days and then hatched in hatching solution for 24 hours. Ten 7 day old F_1_ adult females were randomly selected from each of the weekly cages and were examined for the presence of the *w*Pip *Wolbachia* strain by PCR.

The larvae hatched each week (from 1^st^ to 4^th^ week) from each population were reared to emergence and 50 male and 50 female F_1_ adults were randomly selected and used to establish 6 cage populations per week, as in the previous generation, and eggs were collected as before. The F_2_ eggs, from each one of the weekly F_1_ cages, were collected on weeks 1 and 4, hatched and the larvae reared to emergence. Ten randomly selected F_2_ adult females originating from week 1 and week 4 egg collections were PCR-tested for the presence of the *w*Pip *Wolbachia* strain.

### DNA extraction and PCR amplification

The whole abdomen of individual adult females were dissected and homogenized with 180 μl ATL buffer and 20 μl Proteinase K (QIAGEN, Germany). The homogenates were incubated in a water bath (JULABO, SW-20C, Germany) at 56°C for 3–4 hours, and then DNA extracted according to the procedures of the DNeasy Blood and Tissue Kit (QIAGEN, Germany). The templates were stored at -20°C until use for PCR. The 20 μl PCR reaction consisted of 10 μl PCR Master Mix (QIAGEN, Germany), 1 μl forward and 1 μl reverse primers (10 μM), 1 μl DNA template and 7 μl ddH_2_O. The following *w*Pip phage WO-specific primers were used: *orf*7-cf: CCCACATGAGCCAATGACGTCTG; *orf*7-cr: TTGCTTGCAACACTT ACACTT [34]. The PCR conditions comprised of 5 min at 95°C for the initial denaturation step followed by 35 cycles of 30 s at 95°C, 30 s at 52°C, 45 s at 72°C and 10 min at 72°C for the final extension. PCR products were electrophoresed on a 1.5% agarose gel, which contained 1 μg/ml ethidium bromide. The DNA bands were visualized under UV light (UVP, EC3 Imaging System, USA). Females from the HC colony, both non-irradiated and irradiated, were used as positive controls. The quality of DNA in the samples that tested negative for *w*Pip was measured using primers against the ribosomal *S7* gene of *Ae*. *albopictus* [35,36].

### Statistical analysis

Analysis was conducted using SPSS 13.0 and GraphPad Prism 6.0 softwares. Normality of all the data was examined using the D’Agostino-Pearson omnibus normality test. To analyze emergence rate and female fertility, data were first arcsin transformed. The female emergence rate and ovary length of control and irradiation treatment were compared using one-way ANOVA analysis analysis and Tukey’s post hoc tests for HC strain while using one-way ANOVA analysis for GUA and GT strains. The effects of different irradiation doses on female fertility and fecundity within each strain were also analyzed by one-way ANOVA and Tukey’s post hoc tests.

## Results

### Effect of irradiation on proportion of females laying eggs, female fecundity and fertility

The proportion of females that laid eggs, and female fecundity and fertility of *Ae*. *albopictus* HC, GUA, and GT strains decreased in inverse proportion to the irradiation dose from 0 to 28 Gy (S1 Table and Table 1). A dose of 28 Gy was sufficient to prevent any females laying eggs in both HC and GUA strains (Table 1). The same pattern, that irradiated females did not lay eggs, was also observed in the GT strain when the irradiation dose was at 32 Gy or higher. Even though three eggs were still laid by one GT strain female at 28 Gy, none of them hatched (Table 1). For HC, GUA and GT strains, complete sterility was found when the irradiation dose was 28 Gy or higher (Table 1).

**Table 1:** Effects of irradiation on female fecundity and fertility of *Ae*. *albopictus* HC, GUA and GT strains.

Irradiated strain	Dose (Gy)	N^*^ (♀)	Females that laid eggs	Fecundity (Mean ± SE)	Fertility (%) (Mean ± SE)
**HC**	0	37	37 (100.0%)	49.5 ± 3.7 a	73.7 ± 3.3 (1830) a
	28	24	0	0 b	0 (0) b
	32	16	0	0 b	0 (0) b
	34	25	0	0 b	0 (0) b
	38	16	0	0 b	0 (0) b
	40	24	0	0 b	0 (0) b
**GUA**	0	21	21 (100.0%)	39.7 ± 5.3 A	88.2 ± 3.4 (793) A
	28	20	0	0 B	0 (0) B
	32	27	0	0 B	0 (0) B
	34	26	0	0 B	0 (0) B
	38	19	0	0 B	0 (0) B
	40	20	0	0 B	0 (0) B
**GT**	0	20	20 (100.0%)	58.4 ± 5.6 i	83.4 ± 3.4 (1167) i
	28	16	1 (7.1%)	0.2 ± 0.2 ii	0 (3) ii
	32	14	0	0 ii	0 (0) ii
	34	13	0	0 ii	0 (0) ii
	38	13	0	0 ii	0 (0) ii
	40	14	0	0 ii	0 (0) ii
**HC** ^**#**^	0	49	49 (100.0%)	66.8 ± 3.7 I	78.9 ± 2.7 (3272) I
	23	49	9 (18.6%)	0.6 ± 0.2 II	1.7 ± 1.2 (30) II
	24	75	6 (8.3%)	0.3 ± 0.1 II	1.2 ± 0.9 (21) II
	26	79	1 (1.1%)	0.04 ± 0.04 II	1.3 ± 1.3 (3) II
	28	43	0	0 II	0 II

^#^ Second experiment to determine the minimum irradiation dose required to induce complete sterility in HC strain females after the first experiment showed that HC strain females were completely sterile after exposure to 28 Gy.

* Female which had taken a blood-meal by personal observation.

Within column with the same strain, values followed by different lowercase letters or capital letters or Roman numbers were statistically different (P<0.05) using Tukey’s post hoc tests.

After determining that HC strain females could acquire complete sterility at 28 Gy by irradiating pupae grouped in the centre of a plastic plate (S1B Fig), a follow-up experiment was performed to determine the minimal irradiation dose to completely sterilize HC strain females. However, a few HC strain females exposed to 23, 24 and 26 Gy were still fecund and fertile (Table 1) while 28 Gy induced complete sterility (Table 1).

In light of the above results, sixty HC females irradiated at the pupal stage with 23, 26 or 28 Gy) which did not lay eggs were dissected to ensure they had been inseminated. Fifty-seven out of sixty irradiated HC females (95%) dissected were inseminated (at least one spermathecae was full of sperm) which confirmed that irradiation was the factor responsible for the complete sterility observed.

### Effects of irradiation on female adult emergence rate

The effect of irradiation on the female adult emergence rate was also studied at different irradiation doses (0, 26, 28 and 40 Gy). No significant difference in female adult emergence rate of *Ae*. *albopictus* HC, GUA and GT strains was observed between irradiation treatment and control (HC: F = 1.19, df = 3, P>0.05; GUA: F = 0.89, df = 1, P>0.05; GT: F = 6.00, df = 1, P>0.05) (Table 2).

**Table 2:** Effect of a pupae irradiation dose on female adult emergence and ovary length (Mean ± SE) of *Ae*. *albopictus* HC, GUA, and GT strains.

Dose (Gy)	Emergence rate (%)			Ovary length in mm (N)		
	HC	GUA	GT	HC	GUA	GT
**0**	97.0 ± 1.0 a	97.0 ± 1.3 a	95.2 ± 1.0 a	1.18 ± 0.02 (30) a	1.13 ± 0.02 (30) a	1.13 ± 0.02 (30) a
**26**	99.0 ± 0.7 a	ND	ND	0.79 ± 0.02 (30) b	ND	ND
**28**	97.0 ± 2.7 a	ND	ND	0.79 ± 0.02 (30) b	ND	ND
**40**	94.5 ± 1.3 a	96.0 ± 1.5 a	91.3 ± 0.7 a	0.69 ± 0.02 (30) c	0.65 ± 0.01 (30) b	0.70 ± 0.01 (30) b

N: Number of ovaries measured; ND: not done. Within column, values followed by different lowercase letters were statistically different (P<0.05) using ANOVA analysis and Tukey post hoc tests for HC strain while using ANOVA analysis for GUA and GT strains.

### Effect of irradiation on ovary length

**Fig 2:**
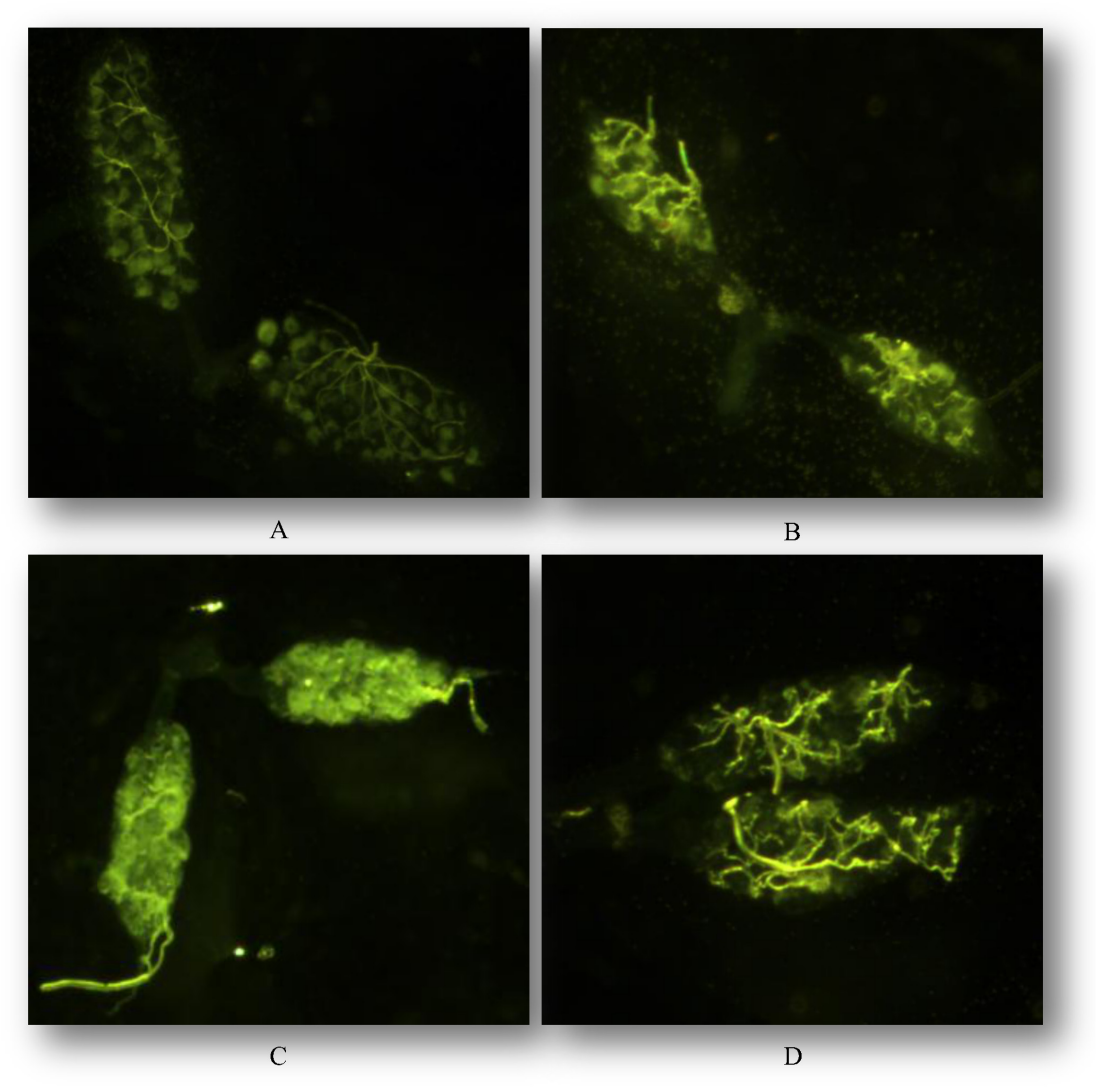
Effect of irradiation on the ovaries of *Aedes albopictus* HC strain. A: Normal egg-follicles in the non-irradiated ovary of the HC strain (30 ×). B, C and D: Gross morphological damage in ovaries of the HC strain after irradiation at 40 Gy (30 ×).

The effect of irradiation on the ovary length and structure was also studied. *Ae*. *albopictus* female exposed to at least 28 Gy did not lay eggs. Microscopic analysis indicated significant differences in the ovary length of control and irradiated females of the HC, GUA and GT strains (HC: F = 136.9, df = 3, P<0.05; GUA: F = 530.0, df = 1, P<0.05; GT: F = 384.5, df = 1, P<0.05) (Table 2). In the HC strain, the ovaries of the 40 Gy treatment were significantly smaller than those of lower dose treatment (26 or 28 Gy) (Table 2). The microscopic analysis also showed gross damage in the structure of the ovaries of irradiated females at 40 Gy of the HC, GUA and GT strains compared to that of the control (Fig 2).

### Ability of irradiated HC females to spread *Wolbachia w*Pip into small cage GUA populations

For both the treatment and control cages, 120 F_1_ adult females (10 randomly selected females from each of the three replicate treatment cages from egg collections on each of the of the 4 consecutive weeks) and 60 F_2_ female adults (10 randomly selected females from each of the three replicate treatment cages from the egg collections of the 1^st^ and 4^th^ weeks) were tested for the presence of the *w*Pip infection status by PCR. All the tested F_1_ and F_2_ females from the treatment cages were negative for the *w*Pip infection (Table 3). However, in the control cages, the average *w*Pip-infection rate ranged from 23.3 to 43.3% in the F_1_ and from 40.0 to 46.7% in the F_2_ females (Table 3). The PCR results of F_1_ and F_2_ indicated that releasing irradiated HC females at a release ratio of 1:5 failed to introduce *w*Pip to the cage population, unlike their non-irradiated counterparts, confirming their sterility after exposure to 28 Gy of radiation and the absence of horizontal transmission.

**Table 3:** Infection rate (Mean ± SE) of *w*Pip *Wolbachia* strain of F_1_ and F_2_ progeny after introduction of irradiated or non-irradiated HC strain females into laboratory cage GUA populations.

Dose (Gy)	F_1_ (%)				F_2_ (%)	
	1^st^ week	2^nd^ week	3^rd^ week	4^th^ week	1^st^ week	4^th^ week
**0**	43.3 ± 8.8	23.3 ± 8.8	30.0 ± 17.3	23.3 ± 3.3	46.7 ± 8.8	40.0 ± 23.1
**28**	0	0	0	0	0	0

## Discussion

In the absence of a robust and efficient sexing system for *Ae*. *albopictus*, we proposed the combination of SIT and IIT to control natural populations of this mosquito species using the triple-infected (*w*AlbA, *w*AlbB, *w*Pip) HC strain [32]. Our previous study showed that the fitness of the HC strain is not significantly different from that of the double-infected (*w*AlbA and *w*AlbB) GUA strain or the aposymbiotic GT strain [32]. In the present study, we determined the lowest irradiation dose (28 Gy) which can confer complete sterility to HC females. Moreover, we tested the scenario of the accidental release of triple-infected HC females, irradiated and non-irradiated, into naturally double-infected GUA populations in laboratory cages. Our results showed that if irradiated at the pupal stage HC females cannot cause invasion of a third *Wolbachia* strain (*w*Pip) into the GUA population. Irradiation thus seems able to prevent a population suppression programme from being converted to population replacement in the case of the inadvertent release of some females alongside the sterile males. This clearly indicates that the integration of SIT with IIT is likely to be a very safe approach to suppression of *Ae*. *albopictus* populations. In addition, another advantage of combining SIT and IIT is the possibility of using 28 Gy for the incompatible males instead of the 40 Gy required for complete sterility of the *Ae*. *albopictus* wild-type males [37–39] and thus minimizing the impact of irradiation on their competitiveness, although needs to be properly investigated in a future study.

Given that the presence of an additional *Wolbachia* strain (*w*Pip) in the HC line with regards to the double-infected natural population of *Ae*. *albopictus* (*w*AlbA and *w*AlbB) could result to a rapid and worldwide replacement of the wild-type population, the HC line should be maintained under very secure conditions to avoid the inadvertent release of triple-infected female individuals. It should be noted, however, that other transinfected lines have been recently constructed which are bidirectionally incompatible with the double-infected population of *Ae*. *albopictus* and therefore population replacement is unlikely to take place if a limited number of fertile females are accidentally released in nature [40,41]. The risk of a new infection to spread under unidirectional or bidirectional CI has been assessed by modelling [42,43]. In the case of unidirectional CI, the frequency of release required for a new infection to spread and become fixed is low unless it has a strong negative effect on fitness. In the case of bidirectional CI, the frequency required for invasion and replacement of the wild population is close to 50%. Interestingly, a recent study in *Culex pipiens* suggests that low dispersal probability and metapopulation dynamics may limit the distribution of two *Wolbachia* strains (*w*Pip11 and *w*Pip31) and the associated CI patterns [44]. It is also important to note that another recent study from the same research group suggests that a *Wolbachia*-based IIT approach may be suitable for the control of *Culex quinquefasciatus* in the islands of the southwestern Indian Ocean [45]).

Applying irradiation at the pupal stage can negatively affect adult emergence and consequently survivorship rates. The current study showed that the application of irradiation doses up to 40 Gy has minimal effects on the emergence rates of HC, GUA and GT female adults (Table 2), and thus no impact would be expected after a 28 Gy dose, making even more beneficial the fact that this lower dose confers complete sterility to HC females. Our results are consistent with previous studies on *Aedes* and *Anopheles* species which observed similarly high adult emergence rates (>90%) after the irradiation of pupae at low doses [8,46,47].

Different mosquito species will require different irradiation doses for inducing complete female sterility. In the present study, it was shown that females from all three strains of *Ae*. *albopictus*, irrespectively of their *Wolbachia* infection status, could be completely sterilized at ≥28 Gy. It was shown that these females were inseminated (ca. 95%) and the lack of egg production was only due to the irradiation treatment and the somatic and germ line cellular damage caused to the ovaries (Fig 2). Although we only followed fecundity and fertility for one gonotrophic cycle, the gross damage caused to the ovaries as well as the results of the population replacement experiments suggest that fertility would not recover with age. It is also worth noting that the length of the ovaries decreased as the irradiation dose increased (Table 2). Similar results have been reported in *Plodia interpunctella*, where the number of developing oocytes of ovaries and the length of ovarioles were decreased after exposure to gamma irradiation above 50 Gy [26].

The spatial heterogeneity of dose distribution in the canister of the X ray machine, especially close to the edge of the plate, which is exposed to a radiation dose about 10% lower than at the centre of the plate [31], may affect the level of induced sterility (as in S1 Table). This effect is seen to some degree with all irradiators, so it is important to conduct dosimetry and calibration exercises before an experimental irradiation. The irradiation method can then be adapted accordingly, for example by centering pupae in the field of exposure where the dose rate is appropriately uniform (S1B Fig) or by increasing the target dose to ensure the minimum dose is adequate. This is even more critical for large scale programmes combining SIT and IIT because the release of any fertile females may cause undesirable population replacement [7–9].

Studies have shown that *Wolbachia* is able to invade uninfected *Ae*. *aegypti* and *Anopheles stephensi* small cage populations completely within seven to eight generations after an initial introduction of infected females at a rate 1:5 [48,49]. In addition, releases of a *w*Mel-infected *Ae*. *aegypti* strain in nature has been able to spread and at the same time provide protection against dengue [50,51]. Similarly, our results show that the *Wolbachia* triple-infection of the *Ae*. *albopictus* HC strain (*w*AlbA,*w*AlbB, *w*Pip) can replace the double-infection of the GUA strain, if HC females are released at an initial introduction infection rate of 1:5 as shown in Table 3. Interestingly, the *w*Pip infection could be detected at a rate of 40.0% to 46.7% in just two generations (Table 3). In contrast, no *w*Pip infection could be detected within the next two generations after the release of irradiated HC strain females (Table 3). This confirms that the irradiation dose at 28 Gy can prevent *Ae*. *albopictus* HC strain females from laying eggs. Taken together, our results suggest that the combination of SIT and IIT provides the best solution to population suppression avoiding the risk of accidental release of fertile *Wolbachia*-infected HC females into GUA populations thus converting population suppression efforts into population replacement. In addition, transinfected strains of *Ae*. *albopictus* have been shown to be resistant to dengue virus [41]. If the same were true of the HC line, then any released irradiated HC females would not only be sterile but also be resistant to the dengue virus.

The combined SIT and IIT strategy to control *Ae*. *albopictus* populations is based on the application of a low irradiation dose (28 Gy) in the release generation to completely sterilize any remaining HC females to prevent population replacement, and inducing complete sterility in males with the combined irradiation and *Wolbachia* infection (*w*Pip). Before this combined approach can be implemented, the potential impact of the low radiation dose on males' mating competitiveness as well as on the expression of *Wolbachia*-induced CI in crosses between triple-infected HC males and double-infected GUA females should be assessed. Both of these aspects are currently under investigation in our laboratory. However, taken together, the data presented here suggest that this approach could be effective.

## Supporting Information

S1 Fig *Aedes albopictus* female pupae were irradiated in two different configurations on the plate. A: Female pupae discretely distributed across the plate; B: Female pupae clustered in the center of the plate. Sterility was induced more uniformly and reliably in the latter case, whereas in the former case differences in dose across the plate led to some individuals receiving a sub-sterilizing dose.
(TIF)

S1 Table Effect of irradiation on female fecundity and fertility of *Aedes albopictus* HC, GUA and GT strains. (DOCX)
