# Supplementary figures and images for: Combining the Sterile Insect Technique with Wolbachia-Based Approaches: II- A Safer Approach to Aedes albopictus Population Suppression Programmes, Designed to Minimize the Consequences of Inadvertent Female Release
Source: PLoS One. 2015 Aug 7;10(8):e0135194. doi: 10.1371/journal.pone.0135194 (PMC4529199; doi:10.1371/journal.pone.0135194)

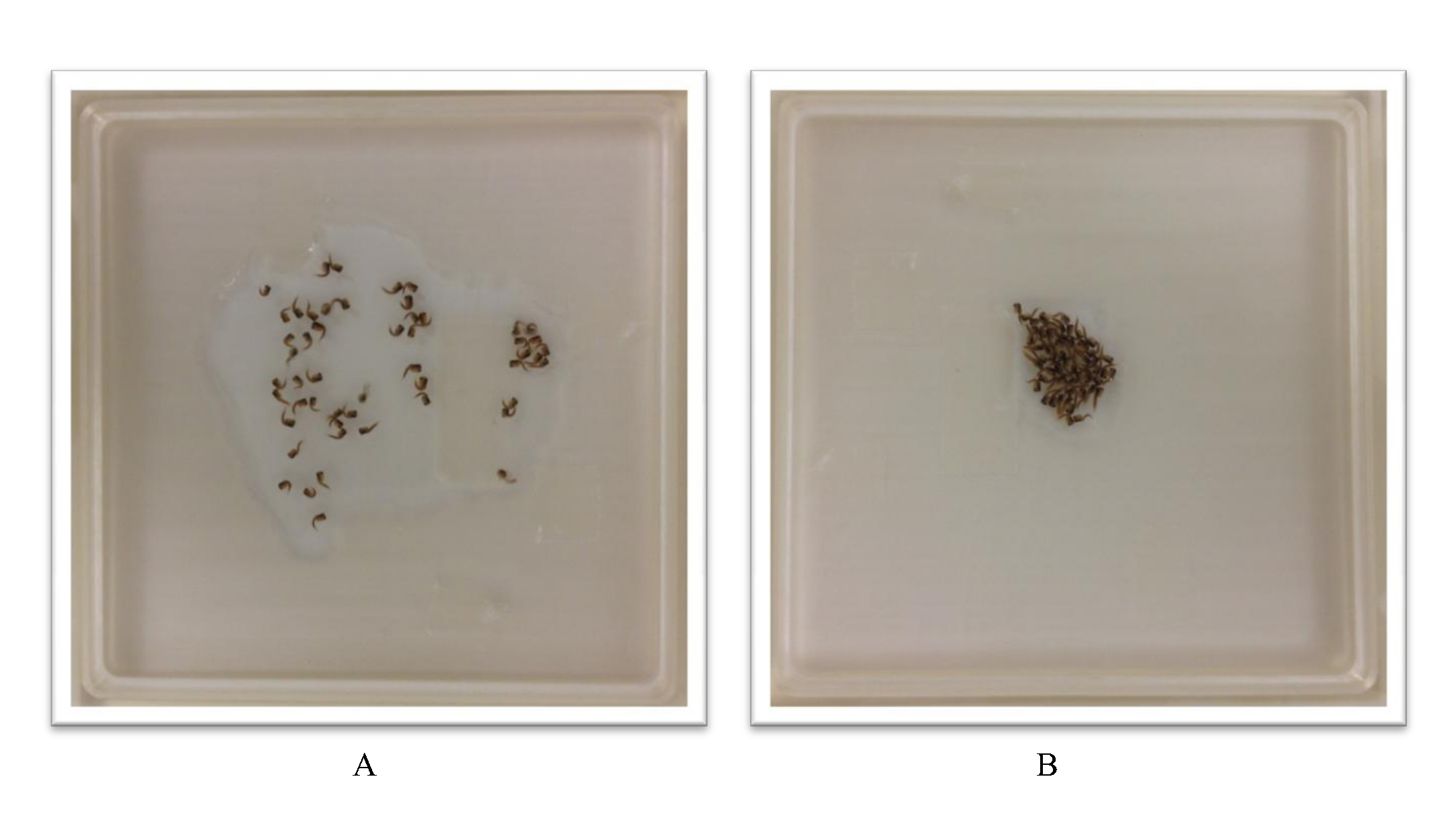

Supplement: S1 Fig — A: Female pupae discretely distributed across the plate; B: Female pupae clustered in the center of the plate. Sterility was induced more uniformly and reliably in the latter case, whereas in the former case differences in dose across the plate led to some individuals receiving a sub-sterilizing dose. (TIF) [file pone.0135194.s001.tif]
