# Supplementary material for: Combining the Sterile Insect Technique with Wolbachia-Based Approaches: II- A Safer Approach to Aedes albopictus Population Suppression Programmes, Designed to Minimize the Consequences of Inadvertent Female Release
Source: PLoS One. 2015 Aug 7;10(8):e0135194. doi: 10.1371/journal.pone.0135194 (PMC4529199; doi:10.1371/journal.pone.0135194)
Supplement: S1 Table — (DOCX) [file pone.0135194.s002.docx]

**S1 Table.** Effect of irradiation on female fecundity and fertility of *Aedes albopictus* HC, GUA and GT strains.

| **Irradiated strain** | **Dose** (Gy) | N^*^ **(♀)** | **Females that laid eggs** | **Fecundity (Mean ± SE)** | **Fertility (%) (Mean ± SE)** |
| --- | --- | --- | --- | --- | --- |
| HC | 0 | 31 | 31 (100.0%) | 37.0 ± 2.1 a | 86.8 ± 2.6 (1148) a |
|  | 11 | 30 | 25 (83.3%) | 22.5 ± 3.0 b | 59.3 ± 7.3 (676) b |
|  | 23 | 31 | 16 (51.6%) | 5.1 ± 1.2 c | 18.6 ± 4.8 (159) c |
|  | 28 | 40 | 3 (7.5%) | 0.1 ± 0.1 d | 3.8 ± 2.8 (4) d |
|  | 34 | 36 | 1 (2.8%) | 0.2 ± 0.2 d | 0.9 ± 0.9 (6) d |
| GUA | 0 | 32 | 32 (100.0%) | 37.5 ± 3.2 A | 87.8 ± 3.7 (1200) A |
|  | 11 | 33 | 31 (93.9%) | 31.5 ± 2.4 B | 76.6 ± 4.4 (1041) B |
|  | 23 | 34 | 9 (26.5%) | 2.3 ± 0.8 C | 11.0 ± 3.8 (78) C |
|  | 28 | 39 | 1 (2.6%) | 1.0 ± 1.0 D | 2.5 ± 2.5 (40) D |
|  | 34 | 43 | 0 | 0 D | 0 (0) D |
| GT | 0 | 19 | 19 (100.0%) | 66.9 ± 3.3 i | 86.1 ± 5.2 (1272) i |
|  | 11 | 19 | 19 (100.0%) | 60.5 ± 5.2 i | 67.1 ± 4.8 (1150) ii |
|  | 23 | 20 | 5 (25.0%) | 1.3 ± 0.7 ii | 3.8 ± 2.3 (28) iii |
|  | 28 | 26 | 1 (3.8%) | 0.3 ± 0.3 iii | 1.6 ± 1.6 (7) iv |
|  | 34 | 21 | 0 | 0 iii | 0 (0) iv |

^*^ Female which had taken a blood-meal by personal observation

Within a column with the same strain, values followed by different lowercase letters or capital letters or Roman numbers were statistically different (P<0.05) using Tukey’s post hoc tests.
